# Periodic Fever, Aphthous Stomatitis, Pharyngitis, and Cervical Adenitis Syndrome (PFAPA): A Clinical Challenge for Primary Care Physicians and Rheumatologists

**DOI:** 10.3389/fped.2019.00277

**Published:** 2019-07-05

**Authors:** Giorgio Costagliola, Giuseppe Maiorino, Rita Consolini

**Affiliations:** Laboratory of Immunology, Division of Pediatrics, Department of Clinical and Experimental Medicine, University of Pisa, Pisa, Italy

**Keywords:** PFAPA, autoinflammatory diseases, diagnostic criteria, children, differential diagnosis, diagnostic delay

## Abstract

**Objective:** To show the different physician's approaches and the difficulties in the diagnosis and management of the Periodic Fever, Aphthous Stomatitis, Pharyngitis, and Cervical Adenitis (PFAPA) syndrome, and to quantify the impact of the disease on the families and on the healthcare system.

**Study Design:** Retrospective analysis on 40 patients diagnosed with PFAPA, focusing on the clinical phenotype, the process of diagnosis, and the management of the febrile episodes. The direct and indirect annual economic cost related to PFAPA in the period preceding the diagnosis were also investigated.

**Results:** The median age of patients at disease onset was 1.75 years and the median time to diagnosis was 14.5 months. During the diagnostic process, only 45% of our patients was firstly addressed to rheumatologic consultation, 32.5% to otorinolaryngologist (ORL), and 22.5% to immunologic consultation. Genetic investigations were performed in the 20% of the cohort. Overall population experienced a median of 60 annual days of fever and, during the critical phase, 40% of patients received more than 5 cycles of antibiotic/year. Seventy five percent required laboratory investigations, 18 (45%) needed to access to emergency department and 15 (37.5%) have been hospitalized. The annual mean direct cost was 1659.5 € for each patient, and the estimated mean indirect cost was 5811.6 € for each parent.

**Conclusion:** Despite a benign clinical course, PFAPA syndrome is associated with a significant impact on the patients, their families and the national healthcare system. PFAPA patients require a large number of medical examinations and laboratory or instrumental investigations during the diagnostic approach and often receive inappropriate treatments. Therefore, we suggest the necessity of a greater awareness and knowledge of the disease among primary care physicians and, finally, of the adoption of more specific diagnostic criteria.

## Introduction

The Periodic Fever, Aphthous Stomatitis, Pharyngitis, and Cervical Adenitis syndrome (PFAPA), firstly described in 1987 by Marshall et al. (1), is the most common among the autoinflammatory diseases of childhood (2), and is featured by recurrent episodes of fever accompanied by at least one of the PFAPA-associated symptoms (aphtosis, pharyngitis, and adenitis). The episodes have a mean duration of 3–6 days in the absence of appropriate treatment, and recur every 2–7 weeks (3–6). Although several works have focused on the definition of new diagnostic criteria (7, 8), the diagnosis of PFAPA syndrome is currently defined according to the modified Marshall's criteria proposed by Thomas et al. (9), which require the presence of recurrent typical febrile episodes in children younger than 5 years old in the absence of evidence of infections or cyclic neutropenia. The recommended treatment of the disease flare is a single dose of prednisone at the dosage of 0.5–2 mg/kg or betamethasone at the dosage of 0.1–0.2 mg/kg, although in some patients a second dose of corticosteroid could be necessary. Colchicine represents the main medical therapeutic option for the reduction of the frequency of the flares, while the indications for tonsillectomy and adenotonsillectomy are still not completely defined: the surgical strategy, however, has showed a beneficial effect on PFAPA syndrome, often permitting the resolution of symptoms (10–12), and therefore has to be considered a favorable option in selected patients.

Despite the increase of scientific contributions to improve the understanding of the clinical features of PFAPA syndrome, the disease is not yet sufficiently recognized (13). Therefore, the diagnostic process and the management of the febrile episodes are often heterogeneous, and require several medical examinations and diagnostic investigations.

The aim of this study is to evidence the main difficulties in the diagnostic process and clinical management of PFAPA patients for both primary care physicians and specialists, and to analyze the impact of the disease, classically defined as “benign,” on the patients, their families and the national healthcare system.

## Methods

The study includes 40 Italian patients (form Tuscany region) diagnosed with PFAPA syndrome between 2016 and 2018, according to the modified Marshall's criteria, followed at the Immuno-Rheumatologic Unit of the University of Pisa. The investigation was retrospectively performed. The clinical phenotype, the disease management by the physicians, the annual number of days of fever and the annual direct and indirect economic costs of the disease, focusing on the period before the formulation of the definitive diagnosis, were analyzed for each patient.

### Clinical Phenotype

We pointed out the clinical manifestations of the febrile episodes, in order to put in evidence the eventual presence of non-specific signs.

### Disease Diagnosis and Management

Concerning the formulation of diagnosis, we focused on the first consultation requested with a medical specialist (rheumatologist, immunologist, and otorinolaryngologist), and the need of a second-level analysis (i.e., molecular analysis for autoinflammatory diseases). The time to diagnosis, since first symptoms, was also reported.

Concerning the management of the single febrile episode, we investigated the execution of hematological routine exams and other investigations (for example, imaging). We documented the need of access to the emergency department and hospitalization. Finally, we reported the inappropriate prescription of antibiotics, individuating patients that received more than 5 cycles of antibiotic/year.

### Impact of the Disease and Economic Costs

The number of annual days of fever and the annual number of days spent for medical consultations out of the febrile episodes were calculated.

Direct costs related to the disease were calculated according to the Diagnosis Related Group (DRG) system (for the episodes of hospitalization) and to the specific schedules published by the Italian Ministry of Health in 2017 (for the other investigations)^1^. Concerning the cost of the antibiotic therapy, we used as a parameter the cost of amoxicillin-clavulanic acid, the most widely used drug in our cohort.

The indirect costs of a single day of work lost by each parent was estimated in 95€, derived from the equation: mean declared income in 2017 (20.940€)/220 days of work (14).

### Disease Outcome

The response to conventional treatment of the flares with corticosteroids and the need of preventive therapy with colchicine or adenotonsillectomy were reported.

### Statistical Analysis

The data on the categorical variables are reported as the percentage and absolute value. The data on the continuous variables with normal distribution (skewness between +1 and −1) are presented as the mean value and standard deviation. In case of asymmetric distribution, the data are presented as median value.

## Results

### Demographic Data and Clinical Phenotype

We included 40 patients (25 males, 15 females), with a median age at disease onset of 1.75 years (age range: 1–8 years). The clinical phenotype, the median duration of single episodes and the interval between episodes are reported in Table 1.

**Table 1:** Clinical features of the 40 patients.

**Median duration of the episode**	**5 days**
**Median Interval between flares**	**30 days**
**Median annual days of fever**	**60 days**
**Fever**	**40/40 (100%)**
**Pharyngitis**	**36/40 (96%)**
**Adenitis**	**21/40 (52.5%)**
**Aphtosis**	**14/40 (35%)**
**Abdominal pain**	**6/40 (15%)**
**Conjunctivitis**	**1/40 (2.5%)**

*The clinical features of our patients are suitable with the current diagnostic criteria*.

By the analysis of comorbidities emerged that 4 patients suffered from febrile seizures, 3 from atopic diseases, 3 from mild IgA deficiency, 1 from myoclonic seizure, 1 from hypothyroidism, and 1 from eosinophilic gastric disease. Concerning auxological parameters, 3 patients showed severe growth restriction (stature <3rd percentile for age and sex) and 1 patient was in the range of obesity.

In 14 patients a positive familial history for recurrent inflammatory episodes of the upper respiratory tract, suitable with PFAPA syndrome, was reported.

### Disease Management

#### Formulation of Diagnosis

The median time to diagnosis, since first symptoms, was 14.5 months. In 18 cases (45%) the first consultation requested by the primary care physician was rheumatologic, with the clinical suspect of PFAPA syndrome, while 13 patients (32.5%) received an otorinolaryngologist (ORL) consultation and 9 (22.5%) an immunologic consultation, with the referral question of “recurrent respiratory infections,” before the rheumatologic assessment. A high number of patients (30 patients, 75%) required immunologic investigations, including the determination of immunoglobulin levels and lymphocyte subpopulations. Moreover, 8 patients (20%) have been investigated for periodic fever-associated genes (MEFV, MVK, TNFRSF1A), and in all the cases the analysis resulted negative.

#### Febrile Episodes

In the period preceding the diagnosis, during the febrile episodes, 30 patients (75%) received first level laboratory investigations (blood cell count, renal and hepatic function, markers of inflammation) with a mean of 1.6 ± 1.9 exams/patient/year; 17 patients (42.5%) received the rapid search for *Streptococcus pyogenes* in pharynx, with a mean of 2.2 ± 2.3 exams/patient/year. During the episodes, 8 patients (18%) required imaging investigations (chest X-ray, abdominal echography, cardiac echography); only 5 patients (12.5%) underwent investigations for specific infectious diseases (serology, blood and urinary culture). Eighteen patients (45%) acceded to emergency department during at least one episode, for the presence of fever or febrile seizures (mean of 1.5 ± 1.3/patient/year) and 15 patients (37.5%) have been hospitalized (median of 5 days of hospitalization).

Sixteen patients (40%) received more than 5 cycles of antibiotic/year during episodes suitable for PFAPA. For 5 out of these patients, the primary care physicians continued to prescribe antibiotics for the treatment of febrile episodes even after the formulation of diagnosis by the rheumatologist.

Table 2 summarizes the investigations performed during the process of diagnosis and the data about the management of the disease flares.

**Table 2:** Investigations during the process of diagnosis and management of the disease flares.

**Process of diagnosis**		**Management of flares**	
**First consultation requested**	**Patients**	**Item**	**Patients**
Immunologic	9	Hospitalization	15
ORL	13	Access to the ED	18
Rheumatologic	18	Standard laboratory exams	30
**Second level investigations**	**Patients**	Search for *S. pyogenes*	17
Genetic exams	8	Serology for infections	5
Immunologic exams	30	Blood culture	2
		Urinary culture	3
		Chest X-ray	3
		Abdomen echography	4
		Echocardiography	1
		Antibiotics (>5/year)	16

*During the process of diagnosis, only 18 patients were promptly addressed to a rheumatologic consultation. During the febrile episodes, several patients acceded to the emergency department or have been hospitalized. ORL, Otorynolaryngologist; ED, Emergency Department*.

### Impact of the Disease and Economic Costs

The median annual number of days of fever was 60, and we reported a median of 2 days spent for medical consultations with specialists out of the febrile episodes. The number of medical consultations in the afebrile period was 74, while the total number of febrile days was 2,373 (95 days spent in hospitalization). Consequently, considering the total of 2,447 days spent because of fever or of medical examinations (mean number of 61,195 days/patient), the estimated maximum indirect cost was 5811.6 € for each parent.

The annual direct cost of healthcare related to PFAPA syndrome, deriving from the previously described medical examinations, diagnostic analyses, hospitalizations and treatments, was 663,818€ in the whole cohort (mean of 1659.5 € for each patient), as showed in Table 3.

**Table 3:** Direct costs related to PFAPA syndrome.

	**Cost (€)**	**Number**	**Total (€)**
Episode of hospitalization	1,660	29	48,140
Access to the ED	25	29	725
Pediatric examination	18.5	508	9,411
Immunologic consultation	18.5	9	166.5
ORL consultation	18.5	19	351.5
Rheumatologic consultation	18.5	46	851
Standard laboratory exams	15	91	1,365
Search for *S. pyogenes*	5.8	37	215
Serology for infectious diseases	9	10	90
Blood culture	11.5	2	23
Urinary culture	8	4	32
Immunologic investigations	38	30	1,140
Genetic investigations	120	24	2,880
Chest X-ray	26	3	78
Abdomen echography	59	4	236
Echocardiography	44	1	44
Antibiotics	7.92	80	633

*The cost of the episodes of hospitalization represents a large percentage of the whole direct cost related to PFAPA syndrome. Moreover, unnecessary investigations and therapy strongly contributed to the final cost. ORL, Otorynolaryngologist; ED, Emergency Department*.

### Disease Outcome

Out of our cohort, most of the patients obtained a satisfactory control of the disease flares with corticosteroids. Three patients required colchicine to reduce the frequency of the disease flares, obtaining in two cases an optimal response. Five patients underwent tonsillectomy: in 3 cases a complete remission was observed, while one patient showed only partial response, and one did not benefit from the surgical treatment. The patients with partial or absent response to tonsillectomy are currently receiving corticosteroids during the febrile episodes.

## Discussion

Despite the existence of approved diagnostic criteria (the modified Marshall's criteria, as discussed in the background section), the diagnosis of PFAPA syndrome still faces considerable difficulties. Therefore, it is often delayed, and its symptoms are frequently misinterpreted as upper respiratory infections, leading to an inappropriate therapeutic strategy. This work evidences the most relevant differences in the approach to the diagnostic process and in the management of the febrile episodes, which involve both the primary care physician and the specialist. The diagnosis of PFAPA disease remains one of exclusion, and consequently patients often undergo several investigations. From the analysis of our cohort emerged that the time to diagnosis has been consistent, and that, in more than half of our patients (55%), the clinical phenotype was firstly interpreted as determined by recurrent respiratory infections, which resulted in ORL or immunologic consultations. On the contrary, only <50% of our patients was referred to a rheumatologic consultation for the presence of periodic fever, remarking that, especially in the first phase of the disease, the clinical phenotype of PFAPA syndrome may not be eloquent. In particular, the periodic pattern of fever is usually recognized tardily, leading to an avoidable diagnostic delay, highlighting the utility of a fever diary in patients with recurrent episodes of fever (15). In absence of a precise diagnosis, almost each febrile episode requires a medical examination, and, as resulted from our analysis, a large part of the patients needed to access to the emergency department (45%) or to be hospitalized (37.5%). This resulted in inappropriate investigations and therapies, which also strongly contributed to the economic costs of the disease on the healthcare system. During the febrile episodes, most of the patients received laboratory investigations, and a considerable percentage received an inappropriate antibiotic treatment. Surprisingly, for some of our patients, the primary care physicians continued to prescribe antibiotic therapy during the disease flares even after the formulation of the diagnosis of PFAPA syndrome by the rheumatologist. Apart from the consideration about the cost of a useless therapy, the use of antibiotics does not help in the resolution of the episode, and contributes to the development of drug-resistance. Even when the patient is addressed to the specialist, the formulation of a definitive diagnosis may require a significant time: in fact, despite the clinical phenotype of PFAPA is sufficiently suggestive, in most of the cases, to differentiate the syndrome from monogenic periodic fever syndromes, particularly Familial Mediterranean fever (FMF), tumor necrosis factor receptor-associated periodic syndrome (TRAPS), and mevalonate kinase deficiency (MKD), the process of exclusion of the monogenic syndromes often leads to an overuse of genetic examinations during the diagnostic process (16). Moreover, in certain circumstances, the use of genetic testing can be justified for differential diagnosis also in the cases of PFAPA displaying a classical picture, if the patient belongs to geographic areas where the rate of FMF carrier is considerable or in the case of incomplete response to adenotonsillectomy (17).

In our cohort of patients, the execution of inappropriate genetic testing was avoided, as patient at low risk of carrying specific mutations were identified according to the Gaslini diagnostic score (16, 18), preventing a relevant increase in the time to diagnosis and in the healthcare costs related to the disease. In addition to our findings, a study by Manthiram et al. (19) reported that, even among rheumatologists, the therapeutic management of the disease flare is heterogeneous, as physicians use different corticosteroid dosage (0.5, 1, and 2 mg/kg, in one or more doses) and timing (introduction at first, second, third day of fever). The same study reports also that the prevention of flares is differently managed among specialists, as rheumatologists are more likely to use colchicine, where infectious disease specialist are more likely to recommend tonsillectomy (19). These different behavioral habits highlight a diffuse variability in the adherence to the current recommendations for the management and the prevention of flares of PFAPA syndrome, only partly depending by the specialization of the physicians, and ascribable to the lack of a behavioral guiding protocol elaborated by the scientific community (20). Despite the benign clinical course of PFAPA syndrome, the disease has a considerable impact on the patients and their families. In fact, in absence of a correct treatment of the disease flare, the patients experience a high number of days of fever, having a potential impairment in their quality of life (21). Additionally, the large number of medical examinations, of laboratory and instrumental investigations, together with the days of work lost by the parents during the febrile episodes, leads a remarkable economic cost for the families and for the healthcare system, particularly in terms of indirect costs, derived by the days of work lost by the parents. Despite the limitations of a single center (Tuscany is recognized as a region with excellent patient care in the Italian scenario) retrospective analysis, our data evidence the difficulties in the recognition of PFAPA syndrome and in the appropriate management of PFAPA patients. Additionally, by comparing the costs of our cohort of patients with those ones derived from common diseases of childhood, emerged that PFAPA patients have a higher economic costs compared to conditions including asthma, atopic disease, recurrent respiratory infections, and celiac disease (22–25). On the contrary, the direct cost for a patient with juvenile idiopathic arthritis (JIA) is markedly higher, as expected (26–28).

The major reason for the delay in the diagnosis and inadequate treatment of PFAPA patients derives from the incomplete knowledge and awareness to this disease by primary care physicians. Therefore, the education of healthcare providers should be crucial to reduce the relevant impact of the disease on the patients, on the families and on the healthcare system, in term of avoidance of inappropriate medicalization and reduction of the number of annual days of fever. Moreover, since current diagnostic criteria have a low specificity (5), we postulate that the adoption of new, more specific criteria, will help to better “identify” the syndrome even among specialists, and to provide a correct, and possibly rapid, diagnosis, contributing in the avoidance of unnecessary examinations and treatments.

## Conclusion

Our work shows that the diagnosis of PFAPA syndrome is often difficult and delayed, and requires several medical examinations and diagnostic investigations, numerous accesses to the emergency or to the hospital and a wide familial involvement. Additionally, the majority of the patients receive an inappropriate antibiotic treatment. Despite the highly benign characteristics of the disease, it has a relevant impact on patients, families and on the healthcare system, in terms of both direct and indirect costs. Therefore, our work suggests the urgent need of a more diffuse knowledge of the disease among primary care providers, and the potential utility of new, more specific criteria, in order to favor a prompter recognition of the syndrome and a consequent approach that limits the amplifications in term of investigations and inadequate treatment.

## Data Availability

The datasets generated for this study are available on request to the corresponding author.

## Author Contributions

GC and GM wrote the paper. RC critically revised the paper. All the authors contributed to the analysis, interpretation of clinical data, read, and approved the final version of the manuscript.

### Conflict of Interest Statement

The authors declare that the research was conducted in the absence of any commercial or financial relationships that could be construed as a potential conflict of interest.

## Footnotes

^1^*Ministero della Salute-Home*. Available online at: http://www.salute.gov.it/portale/home.html

## Abbreviations

PFAPA: Periodic Fever, Aphthous Stomatitis, Pharyngitis, and Cervical Adenitis
ORL: Otorynolaryngologist
FMF: Familial Mediterranean fever
TRAPS: Tumor Necrosis Factor receptor-associated periodic syndrome
MKD: Mevalonate Kinase deficiency.
